# Benchtop NMR in Biomedicine: An Updated Literature Overview

**DOI:** 10.3390/metabo16010003

**Published:** 2025-12-22

**Authors:** Linda Fantato, Maria Salobehaj, Jacopo Patrussi, Gaia Meoni, Alessia Vignoli, Leonardo Tenori

**Affiliations:** 1 Department of Chemistry “Ugo Schiff”, University of Florence, Via della Lastruccia 3-13, 50019 Sesto Fiorentino, Italy; linda.fantato@unifi.it (L.F.); maria.salobehaj@unifi.it (M.S.); jacopo.patrussi@unifi.it (J.P.); gaia.meoni@unifi.it (G.M.); 2 Magnetic Resonance Center (CERM), University of Florence, Via Luigi Sacconi 6, 50019 Sesto Fiorentino, Italy

**Keywords:** NMR spectroscopy, benchtop NMR, metabolomics, lipoproteomics, biomedicine

## Abstract

**Background:** Nuclear Magnetic Resonance (NMR) spectroscopy is a powerful analytical tool in metabolomics, but it is often hindered by the high cost and technical complexity of the machines, limiting its clinical and point-of-care applications. Recent advances in benchtop NMR technology have sought to overcome these barriers by providing more compact, affordable, and user-friendly instruments. This systematic review aims to assess the potential of benchtop NMR in clinical metabolomics, highlighting its practical advantages, current applications, and technological challenges relative to high-field systems. **Methods:** For this systematic review we searched Web of Science and PubMed databases to identify studies employing benchtop NMR spectroscopy in clinical and biomedical applications. The review focuses on works that evaluated metabolic profiling in human and animal disease contexts, compared benchtop and high-field performance, and utilized advanced data analysis methods, including multivariate and machine learning approaches. **Results:** Among the 74 records identified, 15 research articles were eligible, including 11 studies involving human biospecimens and 4 studies concerning animal samples. The selected works were published between 2018 and 2025. These studies demonstrated the potential clinical utility of low-field NMR in differentiating disease states such as tuberculosis, type 2 diabetes, neonatal sepsis, and chronic kidney disease, achieving diagnostic accuracies comparable to high-field instruments. **Conclusions:** Although limited by lower sensitivity and spectral resolution, benchtop NMR represents a significant step toward the democratization of NMR-based metabolomics. Continued hardware development, improved pulse sequences, and the integration of artificial intelligence for spectral processing and modeling are expected to enhance its analytical power and accelerate its clinical adoption.

## 1. Introduction

Metabolomics is an area of omic sciences focused on the qualitative and quantitative analysis of the metabolome, which encompasses the entirety of the metabolites present in a biological system such as a cell, a tissue, an organ, or an entire organism [1]. The primary goal of metabolomic studies is to identify, quantify, and analyze changes in the concentration of the metabolites responsible for biological processes and mechanisms underlying specific behaviors or conditions [2]. Among the several omic sciences, metabolomics is the downstream science in the systems biology information flow. As the information flows from genotype to phenotype, the influence of environmental factors becomes progressively more pronounced. Because the metabolome reflects the dynamic interactions among the genome, transcriptome, proteome, and the environment, metabolomics provide a direct molecular snapshot of an individual’s health or disease state. This makes it a powerful tool in precision medicine for discovering novel biomarkers for disease diagnosis and prognosis, monitoring patients during therapy, and identifying new potential therapeutic targets [3,4,5,6,7,8,9,10,11].

The main two analytical platforms for metabolomics are Nuclear Magnetic Resonance (NMR) spectroscopy and mass spectrometry (MS) [12]. Actually, the two techniques can be considered complementary, with the strengths of each compensating for the weaknesses of the other [8,13,14]. This review focuses exclusively on NMR spectroscopy, which represents our primary area of research.

The standard instrument for metabolomic studies in biomedical applications operates at 600 MHz, which strikes an optimal balance between cost, sensitivity, resolution, and acquisition time [15]. NMR spectroscopy offers several key advantages: NMR is intrinsically reproducible and quantitative over a wide dynamic range, requires minimal sample preparation, and enables the simultaneous detection of several metabolites containing NMR-active nuclei (e.g., up to around 150 and 50 metabolites in urine and serum/plasma samples, respectively) [16]. The main limitations of NMR spectroscopy include its low sensitivity (typically in the micromolar range), high purchase and maintenance costs, and limited portability [17]. In addition, high-field NMR systems require substantial infrastructure, which restricts their suitability for clinical environments and point-of-care applications.

Low-field NMR spectrometers hold promises of a more accessible and portable format that could break down the cost barrier. These spectrometers use permanent magnet-created fields with strengths of several T (^1^H resonance frequency 43–125 MHz); do not require cryogenic gases and, thus, do not need expensive maintenance procedures, nor a Gauss safety line for the magnetic field; occupy reduced footprints that make them suitable for their installation on lab benches or chemical/biological hoods (hence the name “benchtop NMR spectrometers”); and are available at low costs, enabling their application in nearly any setting, including clinical environments [18].

NMR spectroscopy, although intrinsically quantitative, exhibits relatively low sensitivity, which decreases with decreasing magnetic field strength. Moreover, in 1D NMR, resolution linearly scales with the magnetic field strength of the spectrometer, and thus, especially in the crowded spectra of complex biospecimens and at low magnetic field resolutions, is severely degraded, with relevant peak overlaps and strong coupling effects. Nevertheless, recent advances in benchtop NMR have begun to demonstrate its potential in metabolomics, enabling meaningful biochemical insights even outside of high-field laboratory settings and opening new opportunities for biomedical applications. In this systematic review, we focused on recent benchtop NMR applications in different clinical environments, including both human and animal-based studies.

## 2. Methods

The reporting of this review followed the PRISMA (Preferred Reporting Items for Systematic reviews and Meta-Analyses) guidelines to ensure methodological transparency and rigor in the synthesis process [19]. Study selection was conducted according to the criteria outlined below:

Inclusion criteria

▪ Studies that performed metabolomics via low-field NMR on human and animal biofluids.
▪ Studies oriented to clinical applications of low-field NMR.
▪ Scientific articles included in the Web of Science electronic database and/or in the PubMed database.

Exclusion criteria

▪ Not full-length articles, such as case reports, conference proceedings, letters to editor, reviews, and meta-analysis.
▪ Metabolomics performed only using analytical platforms other than low-field NMR (e.g., mass spectrometry and high-field NMR).
▪ Studies analyzing cell models, tissues, or plant- and food-derived samples.
▪ Studies published after 1 September 2025.

The search was conducted in the Web of Science electronic database (https://www.webofscience.com) using the final query of “((TS = (Metabolomic* OR Metabolite* OR Lipoprotein* OR “spectral quantitation” OR “spectral quantification”)) AND TS = (“low-field” OR benchtop OR low-field)) AND TS = (serum OR urine OR saliva OR blood OR feces OR plasma) AND TS = (NMR)” and in the PubMed database (https://pubmed.ncbi.nlm.nih.gov) using the final query of “((Metabolomic*[Title/Abstract] OR Metabolite*[Title/Abstract]) AND (benchtop[Title/Abstract] OR (low-field[Title/Abstract]) OR (“low field”[Title/Abstract]))) AND (NMR[Title/Abstract])”. The search was concluded on 1 September 2025.

Applying the abovementioned inclusion and exclusion criteria, study selection was carried out by screening the titles and abstracts of all publications. Studies that matched the inclusion criteria but had insufficient details in abstracts were further examined by inspecting the complete texts. The procedure was performed independently by two operators.

## 3. Results

**Figure 1:**
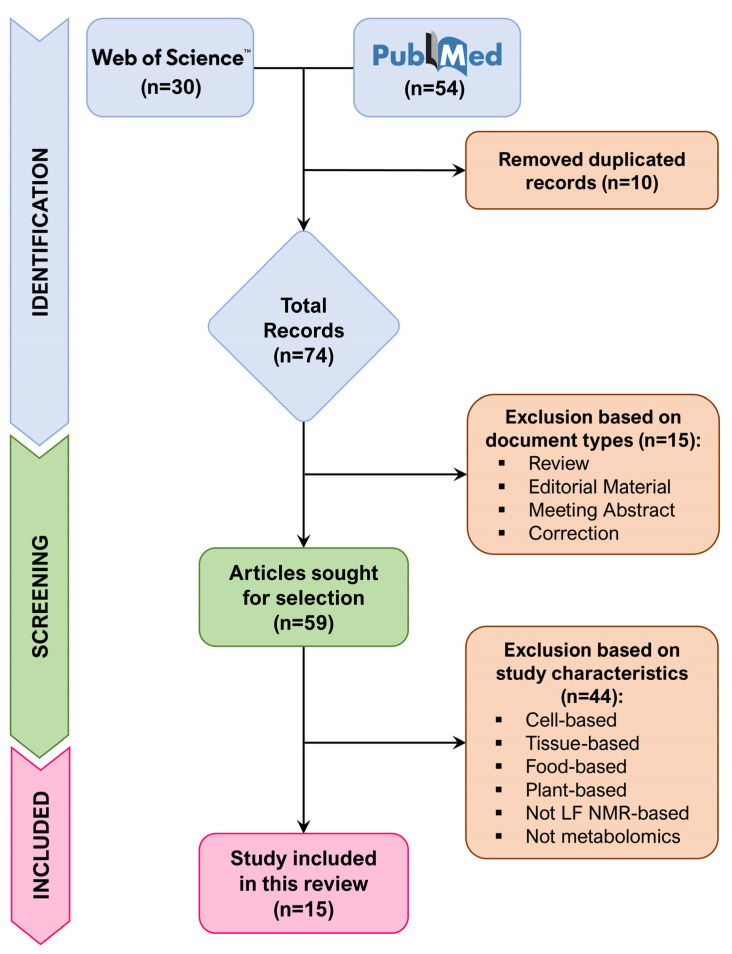
Flowchart of study identification, eligibility, and inclusion.

A total of 15 research articles were identified, including 11 studies involving human biospecimens and 4 studies concerning animal samples (Figure 1). The selected works were all published between 2018 and 2025. The most investigated diseases are tuberculosis and diabetes. A summary of the characteristics of the reviewed studies is provided in Table 1.

### 3.1. Sample Preparation, NMR Acquisition, and Data Analysis Procedures

**Figure 2:**
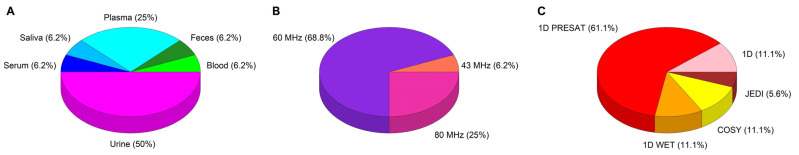
Summary of the main experimental characteristics of the reviewed studies: (**A**) sample types; (**B**) magnetic field (MHz) of instruments; (**C**) NMR experiments.

The procedures utilized for sample preparation, for NMR analysis, and results highlighting significant metabolomic alterations reported in the selected articles are summarized in Table 2. Many of them (8 out of 15) analyze urine samples, followed by studies focused on whole blood, plasma, serum, saliva, and feces (Figure 2A). For the papers that reported sample handling procedures, most samples were stored at −80 °C until NMR acquisition, according to best practice for metabolomics.

A specific, well-defined and standardized protocol for sample preparation prior to NMR analysis at low field is not available. In the majority of studies, biofluids are supplemented with a specific buffer solution, which is used to adjust the internal pH to a standard value of either 7.00 or 7.40. It is generally a phosphate buffer, also containing ^2^H_2_O, 3-(Trimethylsilyl) propionic-2,2,3,3-d_4_ acid (TMSP) as an internal chemical shift reference and sodium azide (NaN_3_), a water-soluble bacteriostatic preservative used to prevent bacterial contamination. However, an exception is noted in the study by Stolz et al., which used methanoic acid as the reference.

Benchtop NMR spectrometers operating at 60 MHz are the most utilized in the reviewed articles; in four studies, 80 MHz spectrometers were utilized and in one study both 60 MHz and 43 MHz spectrometers were used (Figure 2B).

In the majority of the studies, one-dimensional (1D) proton (^1^H) NMR experiments with presaturation (PRESAT) were used. Presaturation is easy to implement and efficiently suppresses the water signal, which is often the dominant component in biofluids. However, water suppression with WET (water suppression enhanced through T1 effects) seems to be particularly suitable for benchtop NMR metabolomics because it effectively removes the dominant water signal without compromising metabolite integrity and metabolite quantitation (Figure 2C). NMR sequences (when reported) and acquisition parameters, such as the number of scans, acquisition time, and relaxation delay, vary across the studies depending on the specific experimental requirements and objectives. Two studies also employ two-dimensional (2D) COSY NMR experiments to increase the depth and resolution of metabolic analysis.

Although the studies analyzed were conducted on different types of biospecimens with a small number of samples, and varied pre-analytical and analytical procedures, some common patterns emerge. These can be interpreted as preliminary recommendations for the use of low-field NMR: sample storage at −80 °C, the use of buffers to stabilize pH (e.g., phosphate buffer), and the application of ^1^H NMR sequences incorporating solvent suppression techniques (e.g., PRESAT/WET).

Across the reviewed studies, data analysis followed a consistent multistep workflow. After spectral processing (including Fourier transformation, phase and baseline correction, and normalization), most authors employed multivariate statistical methods for dimensionality reduction, classification, and univariate analysis for biomarker identification. Principal Component Analysis (PCA) and Partial Least Squares-Discriminant Analysis (PLS-DA) (and PLS-derived methods) represent the most used approaches, indeed only some studies incorporated machine learning techniques such as Random Forest and Support Vector Machine to enhance predictive performances. Statistical significance was assessed through univariate tests (e.g., t-tests, ANOVA, Mann–Whitney, and Kruskal–Wallis) often corrected for multiple comparisons. The most used data analysis platforms are R, MetaboAnalyst, SIMCA, SPSS (version 25, 26 and 27), MATLAB (version 2018b and 2021), and MestReNova. The software Chenomx and the database HMDB were widely used for metabolite identification.

As a general observation, the analytical strategies adopted for low-field NMR data closely mirror those traditionally used in high-field NMR metabolomics, relying on established chemometric and statistical frameworks rather than developing methods tailored to lower magnetic fields. However, the spectral properties of low-field data (e.g., broad signals, peak overlap, and low signal-to-noise ratio) pose relevant challenges for signal assignment and quantitation. These limitations have been mitigated in the analyzed articles by applying a fingerprinting approach rather than a profiling one or by using regression approaches on the basis of high-field data.

### 3.2. Results Described in the Reviewed Studies

This section presents the main findings from the selected studies, organized according to the type of biological fluid analyzed. Particular attention is given to the various biomedical applications of low-field benchtop NMR, highlighting its growing use in the diagnosis, and metabolic characterization of a wide range of pathological conditions.

#### 3.2.1. Human Urine

Human urine serves as a valuable biological fluid for disease diagnosis and metabolic profiling. The literature on metabolomic studies with benchtop NMR offers insights into conditions such as type 2 diabetes (n = 3 studies), tuberculosis (n = 2 studies), and neonatal sepsis (n = 1 study).

**Figure 3:**
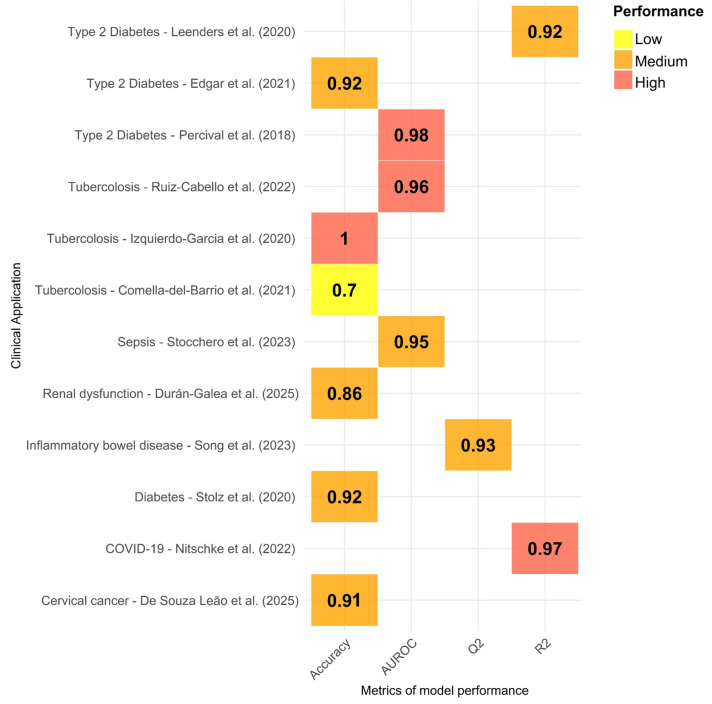
Model performances (reported with different metrics) across the 12 reviewed studies where multivariate statistical analysis was performed. Red: optimal results, orange: discrete results, and yellow: good results. AUROC: area under the receiver operating characteristic curve.

Type 2 diabetes (T2D) accounts for the major number of metabolomic studies applying low-field NMR to urine samples. Percival et al. [20], in their study, presented the first application of benchtop low-field NMR for the rapid and non-invasive metabolic profiling of human urine. Despite resonance overlap challenges, the analytical sensitivity was excellent, with strong discrimination between type 2 diabetes patients and healthy controls with an area under the ROC curve (AUROC) value of 0.975. They successfully identified key urinary metabolites associated with type 2 diabetes: elevated glucose, citrate, creatinine, acetate, acetone, and 3-D-hydroxybutyrate, along with reduced hippurate and indoxyl sulfate in T2D patients. Altered levels of N-acetyl metabolites and lactate were also reported, (Table 2). The potential of this analytical platform as a viable, cost-effective alternative for metabolic screening in diabetes research has been further confirmed by the studies of Leenders et al. [2] and Edgar et al. [22] (Figure 3). In the former, the authors identified a clear cluster separation of T2D and control samples with R^2^ and Q^2^ values of 0.924 and 0.611, respectively. A total of 18 metabolites were identified as significant, and among these, 5 were found to be correlated with urinary glucose levels. Specifically, creatinine, alanine, citrate, lactate, and N-acetylsugar/N-acetylamino acid resonances all showed a negative correlation with urinary glucose. Vice versa, diabetic patients with lower urinary glucose levels exhibited higher levels of hippurate, indoxyl sulfate, citrate, and lactate compared to those with elevated glucose levels. In the latter study, the authors found out that citrate, N-acetylated metabolites, and creatine/creatinine had significantly higher concentrations in T2D urine samples than those of healthy controls subjects. Furthermore, downregulated levels of lactate, hippurate, indoxyl sulfate, and 3-(3-hydroxyphenyl)-3-hydroxypropanoate were also detected in T2D patients.

Izquierdo-Garcia et al. [21] examined tuberculosis (TB) metabolic profiling in the urine of adults using both low-field and high-field NMR. Despite low resolution, PLS-DA models calculated on low-field spectra achieved high accuracy, correctly classifying 87.3% of TB cases against pneumonia and 85.2% against latent tuberculosis infection. Accuracy of 100% was obtained comparing TB with uninfected individuals (Figure 3). Despite LF-NMR’s lower resolution, they were able to discriminate TB patients by identifying the altered concentrations of eight metabolites: aminoadipic acid, citrate, creatine, creatinine, glucose, mannitol, phenylalanine, and hippurate.

Comella-del-Barrio et al. [33], in their study, confirmed the potential of low-field NMR for TB diagnosis and monitoring by identifying a urine NMR-based metabolic fingerprint associated with TB in children. Indeed, they show that TB vs. non-TB groups have different metabolic patterns, but the signal overlapping limits the ability to assign or detect specific metabolites responsible for the discrimination. They performed a PLS-DA model and achieved a performance accuracy of 70% when discriminating presumptive TB from controls.

Stocchero et al. [23] applied benchtop NMR metabolomics to investigate samples from neonates with and without sepsis. In this study, MS and low-field NMR data were analyzed, revealing distinct metabolic profiles between the two groups. The model calculated on low-field NMR data showed comparable results (AUROC = 0.95) to the one calculated on MS data, supporting its utility in neonatal sepsis screening (Figure 3). The authors found an upregulation of several metabolites (2,3,4-trihydroxybutyric acid, 3,4-dihydroxybutanoic acid, D-glucose, gluconate D-serine, hippuric acid, lactate, L-threonine, N-glycine, pseudo uridine, ribitol, kynurenic acid, myo-inositol, taurine, and phenylalanine) in discriminating neonates developing sepsis from the controls at the urinary metabolic level.

#### 3.2.2. Human Saliva

Edgar et al. [24] developed a computational approach to simulate and interpret ^1^H NMR spectra of saliva samples across a range of magnetic field strengths (45–600 MHz), with the aim of bridging high-field and benchtop NMR metabolomics. Using human saliva as a model matrix, they generated simulated spectra for key metabolites based on high-field data and compared them with experimental spectra acquired with a 60 MHz benchtop instrument (Figure 3). Of the 48 resonances visible in the 400 MHz profiles, 19 analytes were detectable at 60 MHz, but only 5 metabolites were quantified (propionate, acetate, methanol, glycine, and formate). Moreover, the authors identified potential macromolecular binding effects in saliva that can influence quantification.

#### 3.2.3. Human Whole Blood

Stoltz et al. [25] evaluated the feasibility of using low-field NMR spectroscopy for glucose quantification in whole blood compared to the standard procedure by enzymatic testing. Challenges included broad spectral lines, glucose signal overlapping with water, and interference from other blood metabolites. The study first tested glucose solutions and bovine plasma before analyzing 117 whole blood samples from an oral glucose tolerance test. Interestingly, mixing samples before analysis minimizes sedimentation and preserves accuracy. Overall, the results show that spectral quantification of glucose in whole blood samples is feasible with an accuracy of 92% (Figure 3), although enzymatic methods remain superior for point-of-care testing. However, benchtop NMR offers a non-invasive alternative for specialized applications, such as quality control of stored blood or continuous monitoring in extracorporeal systems.

#### 3.2.4. Human Serum and Plasma

From an application standpoint, the three identified studies which analyzed blood serum or plasma by low-field NMR span a wide biomedical spectrum: Nitschke et al. [26] target inflammatory responses and COVID-19-related metabolomic alterations, Wist et al. [27] address lipoprotein quantification for future cardiovascular risk assessments, and De Souza Leão et al. [28] focus on cervical cancer detection (Table 1).

The studies of Nitschke et al. [26] and Wist et al. [27] proposed two complementary strategies to addresses the intrinsic limitations of low-field NMR. The first one proposes the use of advanced pulse sequences (i.e., JEDI) to enhance specificity. The authors effectively distinguished SARS-CoV-2-positive patients from control subjects. Patients showed increased levels of acetylated glycoproteins (Glyc) and decreased levels of supramolecular phospholipid composite (SPC) compared to healthy controls. Furthermore, the SPC-to-Glyc ratio was comparable in the 600 MHz and 80 MHz spectra (R^2^ = 0.97). In the second study, regression models trained on high-field data were calculated to predict lipoprotein-related parameters measured at low field. Despite the expected challenges, such as reduced spectral dispersion and lower field strengths, 25 out of 28 major parameters were successfully predicted by the authors, including the primary parameters commonly used as cardiometabolic risk markers (LDL-C, HDL-C, total cholesterol, Apo-A1, Apo-B100, and Apo-B100/Apo-A1).

In the study by De Souza Leão et al. [28] the efficiency of low-field ^1^H NMR spectroscopy was combined with chemometric modeling for the detection of cervical cancer. The metabolomic model achieved an average accuracy of approximately 91%, indicating a robust ability to distinguish the plasma samples of healthy individuals from those of patients with precancerous lesions (high-grade cervical intraepithelial lesions) and with cervical cancer, demonstrating great potential in identifying biochemical patterns associated with disease progression. Specifically, the authors observed increased levels of N-acetylated glycoproteins, citrate, choline, and glycine in patients with precancerous lesions and in the cervical cancer groups. They also highlighted increased levels of LDL, VLDL, α-glucose, and uridine in patients compared to controls.

Taken together, these exploratory, proof-of-concept works illustrate a promising methodological path. They show how benchtop NMR can be used for molecular phenotyping in small-scale studies, paving the way for the future validation of its clinical utility in point-of-care settings in larger cohorts.

#### 3.2.5. Bovine Plasma

Ruiz-Cabello et al. [29] used low-field NMR metabolic fingerprinting to effectively differentiate cows with tuberculosis and paratuberculosis and healthy controls, obtaining a diagnostic AUROC of 0.96 (Figure 3) with a confidence interval of 0.78–1. They reported lower levels of 3-hydroxybutyrate, alanine, creatine, formate, glutamine, isobutyrate, isoleucine, leucine, O-phosphocholine, phenylalanine, tyrosine, valine, and τ-methylhistidine and a higher concentration of glucose in diseased animals with respect to healthy controls. These results highlight low-field NMR as a reliable tool for TB diagnosis in cattle, offering a portable and effective alternative to conventional methods.

#### 3.2.6. Mice Feces

Song et al. [30] using low-field NMR effectively identified metabolic differences in healthy mice and mice affected by inflammatory bowel disease (IBD) induced by dextran sodium sulfate. The fecal metabolic profiles characterized at 60 MHz and 800 MHz showed good comparability, successfully discriminating the induced IBD group from the healthy control with a Q^2^ value of 0.93 (Figure 3). OPLS-DA models calculated on spectra obtained at 60 MHz and associated VIP scores allowed the authors to identify that the levels of butyrate, propionate, isoleucine, valine, leucine, alanine, aspartate, glycerol, and threonine were decreased in the induced IBD group as compared to the control group. Whereas the levels of acetate, succinate, and glucose were increased in the induced IBD group.

#### 3.2.7. Animal Urine

Benchtop NMR was used by Finch et al. [31] to analyze urine samples from a very small cohort of cats (four animals), distinguishing those with chronic kidney disease (CKD) from healthy controls. CKD cases showed altered levels of acetate, creatinine, citrate, taurine, glycine, serine, threonine, hippuric acid, and phenylacetylglycine. These findings suggest that benchtop NMR has the potential to detect metabolic differences in feline urine for non-invasive CKD monitoring. The possibility of diagnosing CDK by low-field NMR has also been investigated by Durán-Galea et al. [32] in dog urine samples (Figure 3). Even in this case, the PLS-DA model achieved high performance, in terms of accuracy (86%), in distinguishing between healthy and Leishmania-infected dogs, between CKD from leishmaniasis, and between CKD from other causes. However, for none of those comparisons any significantly altered metabolite was reported.

## 4. Discussion

NMR spectroscopy has long been a cornerstone of metabolomics due to its ability to provide detailed and reproducible metabolic profiles. While high-field NMR remains the gold standard in terms of sensitivity and spectral resolution, its high cost, large space requirements, and operational complexity have limited its widespread use in clinical and ‘point-of-care’ settings. The emergence of benchtop NMR systems has addressed many of these limitations, offering a more accessible, cost-effective, user-friendly, and portable alternative for metabolomic analysis. Indeed, unlike high-field spectrometers, which require expensive superconducting magnets and cryogenic cooling, benchtop NMR instruments have permanent cryogen-free magnets, significantly reducing initial investment and maintenance costs. This financial accessibility makes benchtop NMR a viable option for smaller laboratories, hospitals, and field-based research facilities that may lack the infrastructure for high-field systems. Furthermore, benchtop NMR systems are small enough to be placed on laboratory workbenches, allowing for real-time metabolic analysis at or near the site of patient care. This portability also has significant implications for decentralized healthcare, enabling rapid and accessible diagnostics in remote or resource-limited areas where conventional NMR technology would be infeasible. This democratization of NMR technology allows a broader range of researchers and clinicians to perform metabolic analyses, thereby accelerating the integration of metabolomics into routine medical practice. Nevertheless, benchtop NMR is not a replacement of high-field machines; indeed, in a research setting, where the aim is to identify the highest possible number of metabolites, high-field NMR instruments are the most suitable choice, whereas in a clinical setting, where the goal is to detect a limited panel of specific biomarkers, a low-field NMR instrument could be the most fitting approach.

From a clinical perspective, even if in its technological infancy, low-field NMR has demonstrated its ability to provide valuable insights into disease diagnosis, monitoring, and biomarker discovery. However, as most of the available studies are preliminary, with limited population size and often being monocentric without independent validation cohorts, the application of low-field NMR in clinical settings is still far from being integrated into routine practice. This review has highlighted multiple studies where benchtop NMR successfully characterized the metabolic profiles of patients with diseases such as tuberculosis, type 2 diabetes, neonatal sepsis, and chronic kidney disease, often with classification accuracies comparable to those of high-field instruments when coupled with advanced statistical and multivariate data analysis techniques.

A key aspect for clinical translation that deserves consideration is reproducibility. It is worth noting that NMR spectroscopy is intrinsically a quantitative and highly reproducible technique, a feature that remains valid even at low magnetic field strengths [34]. Although a systematic assessment of reproducibility across studies is beyond the scope of this review, some indications can nevertheless be inferred from the examined literature. In particular, (a) studies investigating the same biofluid generally report the identification of largely overlapping sets of metabolites, despite differences in experimental design and data analysis strategies. Moreover, (b) two independent studies on type 2 diabetes using urine samples consistently identify a common group of metabolites (i.e., citrate, creatinine, glucose, indoxyl sulfate, hippurate, N-acetyl compounds, and lactate) as being statistically different between healthy subjects and patients, suggesting a certain degree of biological and analytical reproducibility. Further in-depth evaluations of reproducibility are not feasible at present, given the preliminary nature of many available studies and the substantial heterogeneity in terms of instrumentation, sample preparation, cohort size, and statistical approaches. We believe that these limitations should be addressed by future, more standardized investigations, particularly in view of clinical implementation.

However, benchtop NMR is not without limitations. Its lower sensitivity and spectral resolution associated with strong peak overlap and second-order effects, compared to high-field NMR, pose challenges in detecting low-concentration metabolites or in disentangling the complexity of biological mixtures. Indeed, only a restricted set of relatively abundant metabolites can be robustly detected in complex biofluids such as plasma, serum, and urine. Consequently, low-abundance or strongly overlapping resonances may decrease the effective detection and quantification thresholds, increasing the risk of false negatives and limiting sensitivity to more subtle metabolic alterations. Furthermore, a key strategy to overcome extensive peak overlap and matrix effects in protein-rich samples like plasma or serum could include the implementation of tailored pre-analytical protocols, including protein removal.

The abovementioned limitations could also be mitigated by the future availability of stronger permanent magnets or by the combination of low-field NMR with other analytical platforms, such as LC-MS. At the time being, commercially available benchtop NMR systems can reach a practical field of approximately 3 T, corresponding to a proton Larmor frequency around 125 MHz. The only instrument available at this magnetic field on the market proposes a flow system that introduces critical issues such as the risk of dilution, cross-contamination, and difficult sample volume standardization, which are unacceptable in metabolomics where sample integrity and reproducibility are paramount. However, existing materials pose a relevant issue for the further development of permanent magnets at higher magnetic fields.

LC-MS is a powerful and established technique capable of detecting an extended panel of metabolites and is even widely used in hospital settings. Additionally, low-field NMR provides a highly complementary approach that is easy to use and requires minimal sample preparation. Therefore, the two techniques are not mutually exclusive but synergistic. LC-MS delivers unparalleled breadth and sensitivity for a comprehensive metabolic snapshot, while automated low-field NMR offers a rapid, simple, and highly efficient solution for high-throughput analysis. This combination creates a powerful workflow, where low-field NMR can act as a robust first-line screening tool, followed by the more in-depth, specific investigation provided by LC-MS [23]. This combined approach remains considerably less expensive than high-field NMR.

Even if the analytical methodology employed in the reviewed papers is often just a small adaptation of already established procedures, as research progresses, we can expect more optimized pulse sequences, computational post-processing and deconvolution methods, as well as tailored statistical techniques to improve sensitivity, resolution, and accuracy. Furthermore, the integration of state-of-the-art artificial intelligence algorithms into data analysis may further refine low-field data, enhancing modeling capabilities for better diagnostic and/or prognostic clinical models.

## Figures and Tables

**Table 1:** Summary of the demographic characteristics and clinical conditions of the included studies.

Author/Year	Cohort Allocation	Cases/Controls	Age Mean (yrs)	Sex	Disease or Condition	Aim of the Study
Percival et al. (2018) [20]	United Kingdom	T2D (n = 10) and CTR (n = 14).	T2D: 45 CTR: 27	T2D: females (n = 7), males (n = 1), NA (n = 2). CTR: females (n = 9), males (n = 5).	Type 2 diabetes	To present an updated protocol for the analysis of biofluids through compact, benchtop NMR measurements.
Izquierdo-Garcia et al. (2020) [21]	Spain, Germany	Active TB patients (n = 189), pneumococcal pneumonia patients (n = 42), LTBI individuals (n = 61), and uninfected individuals (n = 40). TB patients vs. CTR groups (pneumococcal pneumonia, LTBI, and uninfected). TB, tuberculosis; PnP, pneumococcal pneumonia; LTBI, latent TB infection; Uninfected, individuals without infection.	Entire population: 45.9	males (n = 215), females (n = 117).	Tuberculosis	To identify and characterize a metabolic profile of TB in urine by high-field NMR spectrometry and assess whether the tuberculosis (TB) metabolic profile is also detected by a low-field benchtop NMR spectrometer.
Leenders et al. (2020) [2]	United Kingdom	T2D patients (n = 10) and CTR (n = 15).	NA	NA	Type 2 diabetes	Comparing the accuracies and precision of statistical tools via the acquisition of urinary spectra at both high and low operating frequencies.
Edgar et al. (2021) [22]	United Kingdom	T2D (n = 10) and CTR (n = 14).	T2D: 45 CTR: 27	T2D cohort: females (n = 7), males (n = 3). CTR cohort: females (n = 9), males (n = 5).	Type 2 diabetes	To diagnose rapidly and potential prognostic monitor of T2D in human urine samples.
Comella-del-Barrio et al. (2021) [21]	Haiti	Presumptive TB (n = 62) and CTR (n = 55).	Presumptive TB: 7.28 CTR: 6.45	Presumptive TB: females (n = 25), males (n = 37). CTR: females (n = 24), males (n = 31).	Tuberculosis	To describe a urine ^1^H NMR-based metabolic fingerprint for the diagnosis of TB in children.
Stocchero et al. (2023) [23]	Italy	EOS group (n = 15) and CTR (n = 15).	EOS group: 207 days CTR: 213 days	EOS group: males (n = 7). CTR: males (n = 5).	Sepsis	Urinary metabolic fingerprint generated using a benchtop NMR instrument for the early detection of sepsis in preterm newborns.
Edgar et al. (2022) [24]	United Kingdom	Healthy human participants (n = 12) (of whom six were non-smokers and six were regular ‘mild-to-heavy’ smokers of tobacco cigarettes).	Age range: 21–65	Males (n = 5), females (n = 7).	Methodological	To demonstrate the valuable applications of software options available for the prediction of chemical shift values, coupling patterns, and coupling constants in the ^1^H NMR profiles of analyte molecules.
Stolz et al. (2020) [25]	Germany	Healthy blood donors and diabetic patients. (tot. n = 122).	NA	NA	Diabetes	To explore the capabilities of low-field NMR for measuring glucose concentrations in whole blood.
Nitschke et al. (2022) [26]	Spain	SARS-CoV-2-positive patients (n = 29) CTR (n = 28)	NA	NA	COVID-19	To propose adapted versions of JEDI experiments that could provide comparable diagnostic capabilities.
Wist et al. (2025) [27]	Australia, Mauritius, Spain, Unites States of America, Germany	Healthy/free-living population cohort (N_3_ = 127) from Bruker Biospin GmbH & Co. KG, Germany, a free-living population cohort (N_2_ = 127) from CIC bioGUNE, and a diabetic/obese cohort (N_1_ = 119) from the ANPC Australia which was collected in the Republic of Mauritius.	ANPC Cohort: 52 BioGUNE Cohort: 45 Bruker Cohort: 52	ANPC cohort: males (n = 63); BioGUNE cohort: males (n = 92); Bruker cohort: males (n = 92).	Methodological	To translate NMR lipoprotein analysis from high- to low-field systems.
De Souza Leão et al. (2025) [28]	Brazil	CTR (n = 40); pre-cancer patients (n = 40); and patients with CC (n = 25).	CTR: 38.92 Pre-cancer patients: 40.32 CC patients: 43.56	Only women	Cervical cancer	To evaluate the efficiency of benchtop ^1^H NMR spectral data, combined with chemometric analyses in predicting CC and its high-grade precursor lesions.
Ruiz-Cabello et al. (2022) [29]	Spain	TB (derivation set, n = 11), diagnosed with PTB (n = 10), PTB-vaccinated healthy control (n = 10), and healthy PTB-unvaccinated control (n = 10).	NA	NA	Bovine tuberculosis	Plasma fingerprinting using LF-NMR to differentiate TB subjects from uninfected animals, and paratuberculosis-vaccinated subjects.
Song et al. (2023) [30]	Japan	Six C57BL/6JJcl mice were randomly divided into two groups: the control group and the DSS-induced group (n = 3 per group).	11 weeks	All males (n = 6).	Inflammatory bowel disease	To characterize the alterations in the metabolic profile of fecal extracts obtained from dextran sodium sulfate-induced ulcerative colitis model mice and compared them with the data acquired from high-field NMR (800 MHz).
Finch et al. (2021) [31]	United Kingdom	CTR (n = 2) and CKD (n = 2) IRIS stage 2 cases.	NA	Males (n = 2), females (n = 2).	Chronic kidney disease	Benchtop metabolic profiling technology based on NMR to discriminate between chronic kidney disease case and control samples in a pilot feline cohort.
Durán-Galea et al. (2025) [32]	Spain	CTR dogs (n = 14), dogs with CKD due to leishmaniasis (n = 33), and dogs with CKD without leishmaniasis (n = 8).	Entire population: 4.98	Males (n = 9), females (n = 5).	Chronic kidney disease	To identify distinct patterns in the NMR spectra of urine samples from dogs with chronic kidney disease in canine leishmaniasis, reflecting the underlying metabolic profiles.

T2D: type 2 diabetes; CTR: control; NA: not available; NMR: Nuclear Magnetic Resonance; TB: tuberculosis; LTBI: latent tuberculosis infection; PnP: pneumococcal pneumonia; EOS: early onset sepsis; SARS-CoV-2: severe acute respiratory syndrome coronavirus 2; COVID-19: coronavirus disease of 2019; JEDI: J-edited diffusion and relaxation; CIC bioGUNE: Center for Cooperative Research in Biosciences; ANPC: Australian National Phenome Center; CC: Cervical Cancer; PTB: paratuberculosis; LF: low-field; DSS: dextran sodium sulfate; IBD: inflammatory bowel disease; CKD: chronic kidney disease.

**Table 2:** Detailed experimental characteristics of the included studies.

Author/Year	Biofluids	Sample Storage	Sample Preparation	Benchtop NMR	NMR Experiments	Significant Metabolites ^§^
Percival et al. (2018) [20]	Human urine	−80 °C	thawed at room temperature and centrifuged 450 µL urine sample was supplemented with 50 µL phosphate buffer at pH 7.00 (1.00 mol/L) containing 0.05% (*w*/*v*) sodium azide and 50 µL D_2_0 0.05% (*w*/*v*) TMSP transferred in 5 mm NMR tube	Magritek Spinsolve 60 Ultra spectrometer (60 MHz)	1D ^1^H with PRESAT: 64 scans, acquisition time 6.4 s, repetition time 10 s, pulse angle 90°, relaxation delay 1 s.	↑ Citrate, ↑ creatinine, ↑ acetate, ↑ acetone, ↑ 3-D-hydroxybutyrate, ↑ glucose, ↓ indoxyl sulphate, ↓ hippurate, N-acetyl storage compounds, lactate. Key discriminatory biomarkers identified by multivariate analysis in order of effectiveness: citrate, 3-D- hydroxybutyrate, hippurate, N-acetyl storage compounds, alanine, total bulk glucose, lactate, α-glucose, 3-(3-hydroxyphenyl)-3-hydroxypropanoate, indoxyl sulphate, urea in that order of effectiveness.
Izquierdo-Garcia et al. (2020) [21]	Human urine	−20 °C	thawed at room temperature vortexed for 30 s 400 µL urine sample was supplemented with 250 µL of 0.2 M phosphate buffer containing 0.09% NaN_3_, 0.3 mM TMSP in D_2_O vortexed for 30 s centrifuged at 12,000 *g* for 5 min 600 µL was supernatant transferred in 5 mm NMR tube	Magritek Spinsolve 60 Ultra spectrometer (60 MHz)	1D ^1^H with PRESAT: 64 scans, acquisition time 6.4 s, repetition time 10 s, pulse angle 90°, relaxation delay 1 s.	↑ Aminoadipic acid, ↑ creatine, ↑ mannitol, ↑ phenylalanine, ↓ hippurate ↓ citrate, ↓ creatinine, ↓ glucose.
Leenders et al. (2020) [2]	Human urine	−80 °C	buffered with 90:10% (*v*/*v*) ratio of H_2_O: D_2_0 containing 0.05% (*w*/*v*) TMSP transferred in 5 mm NMR tube	Magritek Spinsolve 60 Ultra spectrometer (60 MHz)	1D ^1^H with PRESAT: 64 scans, acquisition time 6.4 s, repetition time 10 s, pulse angle 90°, relaxation delay 1 s. 2D COSY: 8192 datapoints in F2, 256 points in F1, sweep widths of14 and 46 ppm, respectively; 8 scans for F2, acquisition time 0.16 s (F1) and 1.63 s (F2).	T2D patients presenting higher levels of glucose: ↑ glucose, ↓ citrate, ↓ creatinine, ↓ indoxyl sulfate, ↓ urea, ↓ hippurate. T2D patients presenting lower levels of glucose: ↓ glucose, ↑ citrate, ↑ lactate, ↑ indoxyl sulfate, ↑ hippurate.
Edgar et al. (2021) [22]	Human urine	−80 °C	450 µL urine sample supplemented with 50 µL 1M phosphate-buffer solution at pH 7.00 containing 0.05% (*w*/*v*) sodium azide and D_2_0 containing TMSP homogenized transferred in 5 mm NMR tube	Magritek Spinsolve 60 spectrometer (60 MHz)	1D ^1^H with PRESAT: 64 or 128 scans, acquisition time 6.4 s, repetition time 10 s, and pulse angle 90°.	↑ Citrate, ↑ N-acetylated metabolites, ↑ creatine/creatinine, ↓ lactate, ↓ hippurate, ↓ indoxyl sulfate, ↓ 3-(3-hydroxyphenyl)-3-hydroxypropanoate.
Comella-del-Barrio et al. (2021) [21]	Human urine	−20 °C	400 μL urine mixed with 250 μL deuterated buffer containing 0.2 M phosphate solution in D_2_O at pH 7.4, 0.09% sodium azide and 0.3 mM TMSP transferred 600 µL supernatant in 5 mm NMR tube	Magritek Spinsolve 60 Ultra spectrometer (60 MHz)	1D ^1^H with PRESAT, 64 scans, acquisition time 6.4 s.	NA
Stocchero et al. (2023) [23]	Human urine	−80 °C	thawed at 4 °C and shaken 0.9 mL was added to 0.1 mL potassium phosphate buffer (pH 7.4) containing TMSP and sodium azide homogenized 600 µL was transferred to 5 mm NMR tube	Bruker Fourier 80 spectrometer (80 MHz)	Noesypr1d ^1^H with PRESAT: recovery delay 4 s, acquisition time 5 s, 64 scans, 25 °C.	↑ 2,3,4-trihydroxybutyric acid, ↑ 3,4-dihydroxybutanoic acid, ↑ d-glucose, ↑ d-serine,↑ hippuric acid, ↑ lactate, ↑ L-threonine, ↑ N-glycine, ↑ pseudo uridine, ↑ ribitol, ↑ kynurenic acid, ↑ myo-inositol, ↑ taurine, ↑ phenylalanine, ↑ gluconate.
Edgar et al. (2022) [24]	Human saliva	−80 °C	0.5 mL of saliva sample was supplemented with 0.06 mL of 1 M phosphate buffer at pH 7 containing 0.04% (*w*/*v*) sodium azide and 0.05 mL D_2_O and 0.05% (*w*/*v*) TMSP rotamixed and transferred in 5 mm NMR tube	Magritek Spinsolve 60 Ultra spectrometer (60 MHz)	1D ^1^H with PRESAT: 64/384 scans, acquisition time 6.4 s, repetition time 10/15 s, pulse angle 90°, total experiment time around 15 min.	Acetate, formate, methanol, propionate, and glycine.
Stolz et al. (2020) [25]	Human whole blood	NA	600 μL of whole blood/plasma supplemented with 70 μL of methanoic acid and transferred in 5mm NMR tube vortexed for 60 s before insertion temperature equilibrated for 4 min	Magritek Spinsolve Carbon spectrometer (43 MHz) and Magritek Spinsolve Carbon Ultra spectrometer (60 MHz)	1D ^1^H: repetition time 4.5 s, pulse angle 90°, dwell time 500 μ, 8192 points, 128 averages, and total experiment duration roughly 10 min).	Glucose
Nitschke et al. (2022) [26]	Human plasma	−80 °C	thawed at 4 °C for 2 h centrifuged for 10 min at 13,000 *g* at 4 °C 250 µL plasma mixed with 250 µL phosphate buffer containing 75 mM Na_2_HPO_4_, 2 mM NaN_3_, and 4.6 mM TMSP in H_2_O/D_2_O 4:1, pH 7.4 no buffer dilution for JEDI experiments 500 µL was transferred in 5 mm NMR tube sonicated at room temperature for 5 min	Bruker Fourier 80 spectrometer (80 MHz)	1D ^1^H zg: 1 scan, 16,384 data points, relaxation delay 300.0 s, spectral width 40 ppm, and total experiment time 5 min 2 s. Noesygppr1d with PRESAT: 32 scans, 17646 data points, relaxation delay 4 s, spectral width 40 ppm, tot experiment time 4 min 4 s. JEDI: 256 scans, 3224 data points, relaxation delay 2.5 s, spectral width 20 ppm, tot experiment time 15 min.	↑ Glyc, ↓ SPC
Wist et al. (2025) [27]	Human serum and plasma	NA	thawed at 25 °C 300 μL of serum was mixed with 300 μL of phosphate buffer (75 mM Na_2_HPO_4_, 2 mM NaN_3_, 0.08% TMSP in H_2_O/D_2_O 4:1, pH 7.4 ± 0.1) transferred 600 μL in 5 mm NMR tube	Bruker Fourier 80 spectrometer (80 MHz)	1D experiment with PRESAT (noesypr1d): 96 scans (+4 dummy scans), 23,808 data points, relaxation delay of 4.0 s, mixing time of 50 ms, presaturation of 15 Hz and a spectral width of 30 ppm. For the lipoprotein main fractions, a standard 1D experiment with solvent suppression was acquired with 512 scans and a relaxation delay of 2.0 s. All other parameters were equal to 1D experiments of the serum samples.	A total of 25 out of 28 major lipoprotein-related parameters were successfully predicted with a benchtop NMR spectrometer, including the main parameters that are commonly used (LDL-C, HDL-C, total cholesterol, Apo-A1, Apo-B100, and Apo-B100/Apo-A1) as cardiometabolic risk markers.
De Souza Leão et al. (2025) [28]	Human plasma	−80 °C	thawed at 4 °C vortexed 500 µL of sample (without pretreatment) transferred to the 5 mm NMR tube	Magritek Spinsolve MultiX spectrometer (60 MHz)	^1^H NMR spectra were acquired in the “1D PROTON CDEC” mode using a 90° radiofrequency pulse with: 64 scans, an acquisition time of 3.2 s per scan and a repetition time of 15 s. Both ^13^C decoupling and Nuclear Overhauser Effect (NOE) enhancement were enabled.	↑ Lipids (LDL and VLDL), ↑ lactate, ↑ N-acetylated glycoproteins, ↑ citrate, ↑ choline, ↑ glycine, ↑ α-glucose, ↑ uridine.
Ruiz-Cabello et al. (2022) [29]	Bovine plasma	NA	300 μL of filtered plasma was mixed with 300 μL of deuterated water with 1 mM TMSP transferred 600 µL supernatant in 5 mm NMR tube	Magritek Spinsolve 60 Ultra spectrometer (60 MHz)	1D ^1^H with PRESAT: 64 scans, acquisition time 6.4 s, repetition time 10 s, pulse angle 90°, and relaxation delay 1 s.	↓ 3-Hydroxybutyrate, ↓ alanine, ↓ creatine, ↓ formate,↓ glutamine, ↓ isobutyrate, ↓ isoleucine, ↓ leucine, ↓ O-phosphocholine, ↓ phenylalanine, ↓ tyrosine, ↓ valine, ↓ τ-Methylhistidine, ↑ glucose.
Song et al. (2023) [30]	Mice feces	fecal samples −80 °C, lyophilized and pulverized samples −30 °C	250–300 mg powdered feces were mixed with a 1:4 (*w*/*v*) ratio of phosphate buffer (50 mM sodium phosphate, pH 7.4) containing 0.004% NaN_3_ and 10% D_2_O with 0.5 mM TMSP and 1 mM formate shaken for 15 min and centrifuged at 15,000 rpm for 10 min at 4 °C supernatant was collected ultra-filtered using a 5 kDa cut-off centrifugal filter at 9100× *g* and 4 °C 550 μL of the filtrate was transferred to a 5 mm NMR tube a healthy sample was mixed with 550 μL of phosphate buffer containing 0.5 mM TMSP	Magritek Spinsolve 60 Ultra spectrometer (60 MHz)	1D ^1^H with PRESAT: 128 scans, sweep width of 81 ppm, time-domain size of 32,768, acquisition time of 3.2 s, and a repetition time of 7 s (acquisition + relaxation) 299.65 K.	↓ Butyrate, ↓ propionate, ↓ isoleucine, ↓ valine, ↓ leucine, ↓ alanine, ↓ aspartate, ↓ glycerol, ↓ threonine, ↑ acetate, ↑ succinate, ↑ glucose.
Finch et al. (2021) [31]	Feline urine	−80 °C	thawed and diluted by addition of 20% *v*/*v* deuterium oxide, D_2_O	Oxford Instruments X-Pulse spectrometer (60 MHz)	Acquisition temperature 40 °C. 1D ^1^H with WET: 64/128 scans, 6 s acquisition time, 5 s relaxation delay. 2D COSY: 8 scans of 256 slices.	↓ Acetate, ↓ glycine, ↓ serine, ↓ threonine, ↓ citrate, ↓ taurine, ↑ hippuric acid, ↑ creatinine, ↑ phenylacetylglycine
Durán-Galea et al. (2025) [32]	Canine urine	−80 °C	300 μL were mixed with 200 μL of 177 mM phosphate buffer (pH 7.4) containing 0.09% NaN_3_ and 25 mM formic acid transferred 600 μL in 5 mm NMR tube	Magritek Spinsolve 80 Ultra spectrometer (80 MHz)	1D ^1^H NMR with a WET. Data acquisition parameters included a spectral width of 2500 Hz, 65,536 data points and 128 scans.	NA

^§^ ↑ Higher concentrations in patients. ↓ Lower concentrations in patients. Arrows are reported only for those metabolites whose trends are reported in the original publications. TMSP: 3-(Trimethylsilyl) propionic-2,2,3,3-d4 acid; 1D: one-dimensional; PRESAT: presaturation; Cn: urinary creatinine; NMR: Nuclear Magnetic Resonance; COSY: correlated Spectroscopy; T2D: type 2 diabetes; JEDI: J-edited diffusion and relaxation; Glyc: acetylated glycoproteins; SPC: supramolecular phospholipid composite; LDL-C: low-density lipoprotein cholesterol; HDL-C: high-density lipoprotein cholesterol; Apo-A1: apolipoprotein A1; Apo-B100: apolipoprotein B100; CDEC: carbon decoupling; LDL: low-density lipoprotein; VLDL: very low-density lipoprotein; WET: water suppression enhanced through T1 effects.

## Data Availability

Not applicable.

## Abbreviations

The following abbreviations are used in this manuscript:

NMR	Nuclear Magnetic Resonance
1D	One-Dimensional
2D	Two-Dimensional
ANPC	Australian National Phenome Center
Apo-A1	Apolipoprotein A1
Apo-B100	Apolipoprotein B100
AUROC	Area Under the Receiver Operating Characteristic Curve
CC	Cervical Cancer
CDEC	Carbon Decoupling
CIC bioGUNE	Center for Cooperative Research in Biosciences
CKD	Chronic Kidney Disease
Cn	Urinary Creatinine
COSY	Correlated Spectroscopy
COVID-19	Coronavirus Disease of 2019
CTR	Control
DSS	Dextran Sodium Sulfate
EOS	Early Onset Sepsis
HDL-C	High-Density Lipoprotein Cholesterol
HF	High Field
HMDB	Human Metabolome Database
IBD	Inflammatory Bowel Diseases
IRIS	International Renal Interest Society
JEDI	J-edited diffusion and relaxation
LDL-C	Low-Density Lipoprotein Cholesterol
LF	Low Field
LTBI	Latent Tuberculosis Infection
MS	Mass Spectrometry
NA	Not Available
NOE	Nuclear Overhauser Effect
PCA	Principal Component Analysis
PLS-DA	Partial Least Squares–Discriminant Analysis
PnP	Pneumococcal Pneumonia
PRESAT	Presaturation
PTB	Paratuberculosis
SARS-CoV-2	Severe Acute Respiratory Syndrome Coronavirus 2
SPC	Supramolecular Phospholipid Composite
SPSS	Statistical Package for the Social Sciences
T2D	Type 2 Diabetes
TB	Tuberculosis
TMSP	3-(Trimethylsilyl) propionic-2,2,3,3-d_4_ acid
VLDL	Very Low-Density Lipoprotein
WET	Water Suppression Enhanced Through T1 Effects
